# Disparities in the experience and treatment of dental caries among children aged 9–18 years: the cross-sectional study of Korean National Health and Nutrition Examination Survey (2012–2013)

**DOI:** 10.1186/s12939-016-0377-x

**Published:** 2016-06-07

**Authors:** Juyeong Kim, Young Choi, Sohee Park, Jeong Lim Kim, Tae-Hoon Lee, Kyoung Hee Cho, Eun-Cheol Park

**Affiliations:** Department of Public Health, Graduate School, Yonsei University, Seoul, Republic of Korea; Institute of Health Services Research, Yonsei University, Seoul, Republic of Korea; Department of Biostatistics, Graduate School of Public Health, Yonsei University, Seoul, Republic of Korea; Department of Preventive Medicine, Yonsei University College of Medicine, 50 Yonsei-ro, Seodaemun-gu Seoul, 120-752 Republic of Korea

**Keywords:** Access to oral care, Socioeconomic status, Dental caries, DMFT, Dental public health

## Abstract

**Background:**

The aim of this study is to examine the association between parental socioeconomic status (SES) and the experience as well as treatment of dental caries among children aged 9 to 18 years.

**Methods:**

Data from 1253 children aged 9–18 years from the Korean National Health and Nutrition Examination Survey (2012–2013) were analyzed. Parental socioeconomic status was measured using household income level and maternal educational level. The decayed, missing, and filled teeth (DMFT) index was used to measure experience of dental caries (DMFT ≥ 1). Non-treatment of dental caries was measured according to whether the participants who experienced dental caries used a dental service at a dental clinic to treat caries during the previous year. Logistic regression was used to investigate the association between parental socioeconomic status and the experience of dental caries as well as the association between parental socioeconomic status and the non-treatment of dental caries among children that have experienced caries.

**Results:**

A total of 808 subjects (64.5 %) experienced dental caries among 1253 participants, and 582 of these 808 subjects (72.0 %) did not receive treatment among those having experience of dental caries. Parental socioeconomic status was not associated with experience of dental caries. However, those from low- and middle-income households were less likely to receive treatment than those from high-income households (odds ratio [OR] 2.11 [95 % confidence interval (CI) 1.16–3.86], OR 2.14 [95 % CI 1.27–3.62]). In particular, those from low- and middle-income households who had regular dental checkups were more likely to have untreated caries than those from high-income households (OR 3.58 [95 % CI 1.25–10.24]).

**Conclusions:**

This study demonstrates the parental household income–related disparities in children’s dental health treatment. Efforts should be made to lower financial barriers to dental health services, particularly among those from low-income households, in order to reduce dental health disparities in the treatment of caries in children.

## Background

Socio-economic status (SES) has been identified as a predictor for oral health [1]. Social gradients exist between dental disease and income, education, region and race/ethnicity [2, 3]. According to previous studies, there is a global association between SES and the prevalence of dental disease [4]. Dental disease tends to occur more frequently among individuals with social and/or financial hardships [5]. There is a previous study found that individuals with low income and low education are more likely to have poor oral health than those with a higher level of income and education [6]. This ongoing global problem extends to not only the incidence but also the management of disease [7]. Availability and accessibility to health care services have been reported as important factors in maintaining a good health status for all population levels. In a previous study, individuals’ socioeconomic factors such as the level of household income and education were found to be related to a decrease in availability and accessibility of care [5, 8]. Therefore, low household income and low education could be related to an unavailability and inaccessibility of dental care services. Although dental caries are not a life-threatening disease, the cost of treatment is considerable [9]. The limited capacity of the socially disabled hinders their access to proper and timely dental care, whereas access barriers exacerbate the poor dental health status of those with low SES levels [10].

Dental health disparities pass down to the next generation because children are affected by parental factors [11, 12]. Dental caries are common among children worldwide [13]. Children with a deprived family background have a greater likelihood of having caries, and also tend to have more caries than their peers due to the impact of parental factors [14]. Moreover, oral health is linked to an individual’s general health including low weight gain and poor child growth, because poor dental health affects the ability to eat [15]. According to a previous study, both poor dental health and lack of dental care were shown to be most obvious among United States children from socially disadvantaged family population [10]. Children from poverty-stricken and minority backgrounds are the fastest growing population of children, which indicates that high rates of dental disease will pose a significant societal and financial burden in the near future [10]. If the association between parental SES and the dental health as well as the poor use of dental treatment is maintained, then the inequality in oral health and dental care is projected to remain or increase [16–18].

Although the rapidly growing child population from socially disadvantaged family background [10], and the important of this issue, little has been known about the association between parental socioeconomic status and the experience as well as treatment of dental caries among Korean child population. One study on dental caries have recently emerged [19], but the experience of dental caries were analyzed only in a previous study. Therefore, this study was designed to investigate the impact of parental factors, particularly household income and parental education, on dental health status and access to dental health services in order to determine whether dental disparities in children exist or not. Previous studies reported a strong association between maternal education and children’s dental health [20–22]. Therefore, maternal education level was used to measure parental education level.

This study tested two hypotheses with respect to children’s dental health: (i) that lower parental SES is associated with a higher number of experience of dental caries in children; and (ii) that lower parental SES is associated with decreased treatment of dental caries among children experiencing dental caries. In this study, the connection between the level of parental SES and the number of children experiencing dental caries will be addressed first. Then, the relationship between parental SES and the non-treatment of dental caries among children having experienced dental caries will be investigated.

## Methods

### Data and study population

This study was conducted using secondary data from the 2012–2013 Korea National Health and Nutrition Examination Survey (KNHANES). The KNHANES is a nationally representative survey that uses standardized questionnaires based on an independent rolling sampling survey with a complex survey design to assess the health and nutritional status of the Korean population [23]. The KNHANES includes a health interview, a health examination (i.e., physical examination, clinical measurement, and tests), and a dietary interview. The oral examination of KNHANES was conducted at a mobile examination center by four trained dentists using a dental mirror under a fluorescent light [23]. The 2012–2013 KNHANES included data from 16,076 participants (8058 in 2012 and 8018 in 2013). The current study targeted children aged 9 to 18 years from this population to focus on health disparities in permanent teeth. Participants who were less than 9 or more than 18 years old were excluded from the study. Among 2074 children aged 9–18 years, samples with missing values of household income (*n* = 22), non-treatment of caries (*n* = 210), and missing values from other covariates (subjective oral health status [n = 72], frequency of brushing teeth per day [*n* = 2], private insurance [*n* = 37], type of health insurance [*n* = 16], number of tools used for oral care [*n* = 287]) were excluded. Parents with missing information for education level (*n* = 175) were also excluded. Ultimately, 1253 children within 905 households were included. The Institutional Review Board (IRB) of the Korea Centers for Disease Control and Prevention (KCDC) provided formal ethics approval for the KNHANES data sets (IRB approval number 2012-01EXP-01-2C for 2012 and 2013-07CON-03-4C for 2013).

### Measurements

The current or historical experience of dental caries and the non-treatment of caries among children experiencing caries were chosen as the dependent variables. The experience of dental caries was based on the DMFT (i.e., decayed, missing due to caries, and previously filled teeth) scoring variable from the children’s dental examination survey. The DMFT index is a dental caries diagnostic criteria developed by the WHO [24]. The DMFT index, which is a well-known and significant indicator of the dental health of the individual [25], indicates the number of decayed, missing, and filled teeth in the permanent dentition. This index therefore reflects a combination of untreated and treated caries [2]. DMFT scores were obtained by trained dentists using a dental mirror under a fluorescent light [23]. The value of this index was dichotomized into two categories: current or historical experience of caries (DMFT ≥ 1) or having never experienced caries (DMFT = 0). Access to treatment for dental caries was assessed via a question that asked whether the participants have used a dental service at a dental clinic to treat caries for a year (Yes/No), and it was dichotomized into two categories: treatment of caries or non-treatment of caries.

The independent variables were related to parental SES (household income level and maternal educational level), which was obtained via the survey questionnaire. Previous studies reported a strong association between maternal factors and children’s dental health [20, 21]. Therefore, maternal factors such as education level were used to measure parental SES. Household income level was based on values from the household quartile, which was defined as those who live together and share living-related expenditures. Household income was measured as equalized gross household income per month. The equivalent household income was calculated by dividing the monthly household income by the square root of the number of household members and grouping the results into four quartiles: low (< 710,000 KRW in 2012; < 750,000 KRW in 2013), mid-low (710,000–1,470,000 KRW in 2012; 750,000–1,500,000 KRW in 2013), mid-high (1,480,000–2,410,000 KRW in 2012; 1,510,000–2,460,000 KRW in 2013), and high (KRW ≥ 2,420,000 in 2012; KRW ≥ 2,470,000 in 2013). In our study, the low and mid-low groups were combined into one level (i.e., a single low-income household level), and the mid-high household income level was used as the middle-income household level. Therefore, three levels of household income (high, middle, and low) were analyzed in this study. For maternal education level, the highest level of education of the mother of the participant was used. Parental education level had three categories: elementary school graduate or lower, middle or high school graduate, and some college/university or higher.

Several covariates were used for adjustment. The demographic covariates in this study included sex, age, year, region of residence (urban/rural), type of health insurance (NHI or Medical Aid), and private insurance (yes or no). Several confounding variables that were related to dental health behaviors were also included: self-rated dental health status (high, medium, low); previous experience with preventive dental care in the past year, including sealant, fluorine coating, and dental scaling (yes or no); frequency of daily teeth brushing per day (0–1 or ≥ 2); number of tools used for oral care (0 or ≥ 1); and DMFT score (0 or ≥ 1). To determine the frequency of regular dental check-ups, participants were asked the following question: “Did you have a regular dental check-up within 1 year prior to the interview? (yes or no).” We defined the term “regular dental check-up” as a visit to a dental office more than once a year for the purpose of sustaining a healthy oral state despite the absence of an apparent dental problem. This definition of a regular dental check-up was also used in the previous study [26].

### Statistical analysis

We used a chi-squared test for binary variables and the Wilcoxon Mann–Whitney U test for ordinary variables to calculate the frequency and proportion of each variable. A logistic regression was conducted on the weighted data to generate one model containing all the variables of interest as well as potential covariates. Two logistic regressions were conducted to test each of the two hypotheses in this study. The first logistic regression was conducted on the full dataset to determine the association between parental SES and the experience of caries in their child. The second regression was conducted to determine the association between parental SES and the non-treatment of caries among the children who have experienced dental caries (DMFT ≥ 1). All analyses were conducted in SAS Version 9.3 (SAS Institute, Inc., Cary, NC, USA).

## Results

Table 1 summarizes the general characteristics of the children included in this study. A total of 1,253 participants were included in the initial dataset. Of these participants (*n* = 1,253), 808 (64.5 %) had experienced dental caries, and 445 (35.5 %) had not experienced dental caries. Among those who experienced caries (*n* = 808), 582 (72.0 %) did not receive treatment for their dental caries, and 226 (28.0 %) received treatment for their dental caries. The 1253 participants were divided into low (34.4 %), middle (31.8 %), and high (33.8 %) income households, respectively. Those who having experience of caries (*P* = 0.976) was shown to be 64.7 %, 64.7 %, and 64.1 % in low-, middle-, and high-income households, respectively. The non-treatment of caries among children having experienced caries (*P* = 0.005) was shown to be 77.8 %, 72.9 %, and 65.3 % in low-, middle-, and high-income households, respectively. Both experience and non-treatment of caries among children who experienced caries were most frequent in low- and middle-income households and least frequent in high-income households. Most children in this population had mothers with a middle- and high-school graduate education level (61.1 %).

**Table 1:** General characteristics and health behaviors of children aged 9–18 years with incidents and non-treatment of caries

Variables	*N*	%^c^	Experience of caries^a^					Non-treatment^b^ (DMFT ≥ 1)				
			(*n* = 1253)					(*n* = 808)				
			Yes (DMFT ≥ 1)		No (DMFT = 0)			Yes		No		
			*N*	%^d^	*N*	%^d^	*P*	*N*	%^e^	*N*	%^e^	*P*
At the household level (1,253 children in 905 households)	1253	100	808	64.5	445	35.5		582	72.0	226	28.0	
Household income level												
Low	431	34.4	279	64.7	152	35.3	0.976	217	77.8	62	22.2	0.005
Middle	399	31.8	258	64.7	141	35.3		188	72.9	70	27.1	
High	423	33.8	271	64.1	152	35.9		177	65.3	94	34.7	
Maternal education level												
Elementary school graduate or lower	37	3	26	70.3	11	29.7	0.022	22	84.6	4	15.4	0.114
Middle or high school graduate	766	61.1	514	67.1	252	32.9		377	73.4	137	26.7	
Some college or university graduate	450	35.9	268	59.6	182	40.4		183	68.3	85	31.7	
At the children level												
Sex												
Male	668	53.3	387	57.9	281	42.1	< .0001	304	78.6	83	21.5	0.002
Female	585	46.7	421	52.1	164	28.0		278	66.0	143	34.0	
Age												
9 to 12 years	432	34.5	200	46.3	232	53.7	< .0001	131	65.5	69	34.5	0.056
13 to 15 years	445	35.5	310	69.7	135	30.3		232	74.8	78	25.2	
16 to 18 years	376	30	298	79.3	78	20.7		219	73.5	79	26.5	
Region of residence												
Urban	1028	82	665	64.7	363	35.3	0.810	488	73.4	177	26.6	0.388
Rural	225	18	143	63.6	82	36.4		94	65.7	49	34.3	
Type of health insurance												
NHI	1214	96.9	779	64.2	435	35.8	0.133	561	72.0	218	28.0	0.272
Medical aid	39	3.1	29	74.4	10	25.6		21	72.4	8	27.6	
Private insurance												
Yes	1104	88.1	696	63.0	408	37.0	0.003	497	71.4	199	28.6	0.713
No	149	11.9	112	75.2	37	24.8		85	75.9	27	24.1	
Self-rated perception of dental health status												
High	247	19.7	132	53.4	115	46.6	< .0001	100	75.8	32	24.2	0.580
Middle	733	58.5	466	63.6	267	36.4		332	71.2	134	28.8	
Low	273	21.8	210	76.9	63	23.1		150	71.4	60	28.6	
Experience of preventive dental care in the past year												
Yes	121	9.7	73	60.3	48	39.7	0.882	42	57.5	31	42.5	0.011
No	1132	90.3	735	64.9	397	35.1		540	73.5	195	26.5	
Frequency of teeth brushing per day												
0-1	109	8.7	70	64.2	39	35.8	0.892	61	87.1	9	12.9	0.007
2 ≤	1144	91.3	738	64.5	406	35.5		521	70.6	217	29.4	
Number of tools used for oral care												
None	864	69	532	61.6	332	38.4	0.009	387	72.7	145	27.3	0.143
One or more	389	31.1	276	71.0	113	29.1		195	70.7	81	29.4	
Regular checkups for oral care												
Yes	597	47.7	382	64.0	215	36.0	0.906	227	59.4	155	40.6	< .0001
No	656	52.4	426	64.9	230	35.1		355	83.3	71	16.7	
DMFT score												
0	445	35.5	0	0.0	445	100.0	< .0001					
1≤	808	64.5	808	100.0	0	0.0		582	72.0	226	28.0	< .0001
Year												
2012	509	40.6	369	72.5	140	27.5	< .0001	266	72.1	103	27.9	0.406
2013	744	59.4	439	59.0	305	41.0		316	72.0	123	28.0	

^a^Represents the distribution of those with experience of dental caries (DMFT ≤ 1), based on a total of 1253 children

^b^Represents the distribution of those who did not receive treatment of dental caries among those with experience of dental caries (DMFT ≤ 1), based on a total of 808 children

^c^Weighted column percent (%) based on 1253 participants

^d^Weighted row percent (%) of experience of caries (DMFT ≥ 1) based on 1253 participants

^e^Weighted row percent (%) of non-treatment of caries based on 808 participants with experience of caries (DMFT ≥ 1)

Table 2 presents the results from the logistic regression analysis. Several other factors were associated with the experience of caries: female sex (OR 1.91 [95 % CI 1.41–2.59]) and low self-rated dental health status (OR 2.95 [95 % CI 1.77–4.92]). Low and middle-income household was strongly associated with the non-treatment of caries among children experiencing caries (low, OR 2.11 [95 % CI 1.16–3.86]; middle OR 2.14 [95 % CI 1.27–3.62]). In addition, the non-treatment of caries was associated with female sex (OR 0.65 [95 % CI 0.45–0.95]); brushing teeth less than once per day (OR 2.76 [95 % CI 1.13–6.71]); and irregular dental checkups (OR 3.76 [95 % CI 2.58–5.49]).

**Table 2:** Odds ratios for factors associated with the incidents and non-treatment of caries

Variables	DMFT ≥ 1			Non-treatment (DMFT ≥ 1)		
	(*N* = 1253)			(*N* = 808)		
	OR	95 % CI		OR	95 % CI	
Household income level						
Low	0.96	0.63	1.47	2.11	1.16	3.86
Middle	0.95	0.64	1.40	2.14	1.27	3.62
High	1.00			1.00		
Maternal education level						
Elementary school graduate or lower	1.15	0.55	2.40	2.59	0.67	10.00
Middle or high school graduate	1.34	0.99	1.81	0.99	0.61	1.59
Some college or university graduate	1.00			1.00		
Sex						
Male	1.00			1.00		
Female	1.91	1.41	2.59	0.65	0.45	0.95
Age						
9 to 12 years	1.00			1.00		
13 to 15 years	2.47	1.79	3.43	1.61	0.91	2.83
16 to 18 years	3.63	2.44	5.41	2.02	1.10	3.73
Region of residence						
Urban	1.00			1.00		
Rural	0.95	0.56	1.60	0.70	0.41	1.20
Type of health insurance						
NHI	1.00			1.00		
Medical aid	1.39	0.61	3.17	0.43	0.18	1.03
Private insurance						
Yes	1.00			1.00		
No	1.69	1.02	2.82	1.05	0.55	2.02
Self-rated perception of dental health status						
High	1.00			1.00		
Middle	1.81	1.26	2.59	0.83	0.48	1.46
Low	2.95	1.77	4.92	0.87	0.45	1.67
Experience of preventive dental care in the past year						
Yes	1.00			1.00		
No	0.98	0.60	1.59	1.20	0.64	2.22
Frequency of teeth brushing per day						
0–1	1.10	0.67	1.81	2.76	1.13	6.71
≥ 2	1.00			1.00		
Number of tools used for oral care						
0	0.84	0.63	1.12	0.98	0.66	1.46
≥ 1	1.00			1.00		
Regular checkups for oral care						
Yes	1.00			1.00		
No	0.78	0.57	1.07	3.76	2.58	5.49
DMFT score						
1 to 2				1.00		
3 to 4				0.98	0.58	1.65
5 to 6				0.47	0.27	0.83
7≤				0.32	0.17	0.60
Year						
2012	1.00			1.00		
2013	0.72	0.50	1.02	1.28	0.81	2.00

*DMFT* decayed missing and filled teeth, *OR* odds ratio, *CI* confidence interval

Table 3 shows a subgroup analysis of gender and regular dental checkups with respect to the household income level. A stepwise trend by income level was found for each variable, but it was only statistically significant with respect to regular dental checkups. A negative association was found between the household income level and non-treatment of caries among children experiencing caries (OR 3.58 [95 % CI 1.25–10.24]) irrespective of regular dental checkups.

**Table 3:** Odds ratios for untreated children aged 9–18 years according to household income

Variables		DMFT ≥ 1			Non-treatment (DMFT ≥ 1)		
		aOR^a^	95 % CI		aOR^a^	95 % CI	
Gender							
Male	Low	1.47	0.62	3.48	2.46	1.08	5.61
	Middle	0.86	0.55	1.34	2.01	0.92	4.39
	High	1.00			1.00		
Female	Low	1.57	0.50	4.92	1.68	0.86	3.31
	Middle	0.98	0.57	1.68	2.15	1.18	3.92
	High	1.00			1.00		
Regular checkups for oral care							
Yes	Low	1.87	0.71	4.95	3.58	1.25	10.24
	Middle	1.21	0.75	1.95	2.26	1.37	3.71
	High	1.00			1.00		
No	Low	1.19	0.52	2.74	1.74	0.32	9.60
	Middle	0.74	0.45	1.21	1.65	0.83	3.27
	High	1.00			1.00		

*DMFT* decayed missing and filled teeth, *aOR* adjusted odds ratio, *CI* confidence interval

^a^Adjusted for age, year, private insurance, satisfaction of dental health care, frequency of teeth brushing teeth per day, experience of preventive dental care in the past year, number of tools used for oral care, and DMFT score

## Discussion

This study examined two associations: (i) between parental SES and the experience of dental caries and (ii) between parental SES and non-treatment of dental caries in those children having experienced dental caries. Parental factors were assumed to be associated with the experience and treatment of caries during childhood [27].

The findings from this study, however, found only partial associations between parental SES factors and dental health inequalities. In particular, parental SES factors appeared to influence the non-treatment but not the experience of dental caries. The household income level was the sole factor in this association. Although previous studies have linked children’s dental health status and maternal education level, no statistically significant association was observed in this study [15, 20]. The findings of this study therefore suggest that children who have experienced dental caries in low- and middle-income households are more likely to be associated with non-treatment than those from high-income households. In particular, those from low- and middle-income households who had regular dental checkups were more likely to have untreated caries than those from high-income households.

The results of our study did not indicate any significant associations between parental SES, particularly lower household income and lower parental education level, and children’s experience of caries. However, several previous studies did find associations between parental SES and experience of dental caries [5, 10, 19, 28, 29]. In studies in the United states and Brazil, both lower household income and lower education level of the caregiver are likely associated with experience of dental caries among children [5, 28]. Moreover, lower household income was associated with experience of dental caries among children in another study in the US as well as a domestic study [10, 19]. In a study in Japan, a longer maternal education period was related to a decreased risk of dental caries among children, although there was no association with household income [29]. There are some differences between our study and these previous studies that may explain this discrepancy. First, as the two different dependent variables cover different lengths of time period, this may have led to unalike results. One previous study based on the same KNHANES data reported lower household income was related to experience of dental caries among children. In the study, the number of caries at the moment of the survey were used as the dependent variable rather than including previous occurrences [19], which may explain the discrepancy in findings between these two studies. The DMFT score in the current study covered a longer time period due to the inclusion of past and present caries and included the current treatment and disease status, the dependent variable in our study may have been affected by other factors. Parental SES which has changed during the period of growth could affected as well. In addition, there has been Korean government-funded efforts to reduce the incidents of caries among children via the promotion of preventive dental health such as community water fluoridation and dental health education by community health centers [19, 30], which may reduce the impact of parental SES on the incidence of dental caries. Fortunately, there has been a global movement to improve children’s dental health [9].

Household income level likely contributed partially to the relationship between parental SES and non-treatment among children experiencing dental caries. This study found that children in low- and middle-income level households were more likely to not have received treatment of dental caries than those with high-household incomes. This finding was consistent with previous studies in the US and Korea [5, 10, 31] and can be explained by the healthcare policy in Korea. Although universal health coverage through the Korean National Health Insurance (KNHI) was designed to reduce the gap between socioeconomic classes in terms of accessibility and availability to healthcare services, the limited benefit coverage of the KNHI is known to be a problem, causing high economic burden to low-income patients [32, 33]. The limited benefit coverage is mainly due to the high percentage of out-of-pocket payment and uncovered medical services [33]. The high costs of out-of-pocket spending for medical services are a relatively greater burden to low-income households in terms of affordability. According to a previous study, economic burden due to spending costs for dental care was the most common concern among low-income groups who paid out-of-pocket [34]. In addition, the proportion of uncovered medical services is particularly large for Korean dental care services, resulting in severe economic disparities in the use of dental services [35]. Due to these factors, children from low- and middle-income households were associated with non-treatment of dental caries due to the economic burden from the use of medical services. This important finding highlights household income–related disparities in access to dental care services among children and also indicates the existence of a financial barrier to dental care services.

This study also assessed the impact of other factors, such as demographic characteristics and dental health behaviors, on the experience and non-treatment of dental caries. This study found that female children were more likely than male children to have dental caries; however, male children were more likely to experience non-treatment of dental caries than female children. This result suggests that females care more about their dental health than males despite the higher incidence of dental caries among females. No characteristics of dental health behavior were significantly associated with the incidents of dental caries except self-rated dental health status, which was negatively correlated with the incidents of dental caries in agreement with a previous study showing the association between low self-rated dental health status and more experience of caries [6, 36]. This result suggests that more experience of caries can be associated with low self-rated dental health due to the tooth pain from current or previous caries. Although self-rated dental health status was not associated with the non-treatment of caries [37], several factors were significantly negatively associated with the non-treatment of dental caries including the frequency of tooth brushing and regular dental checkups. Our study found that infrequent brushing were more likely to be associated with non-treatment of caries, which is consistent with the finding of the previous study in Mexico [38]. This finding suggests that unhealthy dental care behaviors including infrequent tooth brushing among those having experience of caries can more easily lead to non-treatment of caries. In particular, our study found that children who did not have regular dental checkups were more likely to be related with non-treatment of dental caries, which is consistent with the findings of previous studies [11, 36].

In addition, our study found that children in low- and middle-income households who had regular dental checkups were more likely to not receive treatment than those in high-income households. In previous study, regular dental checkups were found to be a beneficial factor for professional diagnostic and prophylactic services, which are important for dental health [14, 39]. Therefore, children who had regular dental checkups also had information about their dental status based on diagnostic results, and they were able to make a clear decision regarding treatment before the disease deteriorated [14, 40]. On the other hand, those who did not have regular dental checkups may not have been able to acknowledge whether they had any caries or not. However, our findings showed that although children who had regular dental checkups were more clearly aware of their medical needs, there was nevertheless a household income–related disparity in the treatment of caries. Our findings show that low-income children are more likely to not receive treatment of caries due to financial barriers even when children have already recognized that they need to receive treatment. Similarly, the lack of association between regular dental checkups and treatment of caries suggests that the current dental care services are not effective enough to lessen the financial barriers even for those with clear medical needs.

There are several limitations to this study. First, this study is based on cross-sectional data, which reduces the ability to determine an association between the factors and the experience and non-treatment of dental caries. Second, this study did not address the impact of paternal factors. Although the original study intended to use information from both parents, a significant amount of paternal information was missing from the data. Third, there was an association found between the self-rated perception of dental health status and untreated caries. A further study should be conducted to investigate the association between low self-rated health and untreated caries. Finally, this study did not analyze certain characteristic variables that are observed only in students, such as variables that address the length of time staying at school and the significant pressure to study faced by Korean students. A future study should consider such confounding variables to determine the influence on dental health, such as the stress associated with studying and the number of hours during which students are engaged in school activities.

This study reveals several important policy implications. First, the financial barriers to obtain restorative dental health services need to be eliminated. There exists an invisible barrier that prevents children from low-income households from receiving treatment. It may be more difficult for low-income households to afford the financial burden of children’s dental treatment, which may represent a significant proportion of the medical expenditure from their limited household income. The most effective policy would be a coverage increase in dental services. According to a previous study in Korea, the lack of coverage has resulted in access barriers and dental health disparities, and the wealthy receive the most advantage from the dental health gradient [41]. Second, the distribution of resources for dental services should focus more on the low-income households. Third, the lack of association between regular dental checkups and treatment of caries highlights the need for effective dental care, particularly for low-income household children. The need for dental health services increases as the level of household income decreases. Therefore, supportive policies with proper intervention can reduce the gap in dental care access between the high- and low-income households [1, 5, 10, 25].

## Conclusion

This study revealed that parental SES-related inequalities were associated with the treatment of dental caries. In particular, those from low- and middle-income households who had regular dental checkups were more likely to have untreated caries than those from high-income households. There is a need to reduce household income-related disparities in children’s dental health treatment. Through the findings presented here, we hope to contribute to the establishment of a dental health policy that reduces household income-related inequalities in dental care.
